# Influences of Ketogenic Diet on Body Fat Percentage, Respiratory Exchange Rate, and Total Cholesterol in Athletes: A Systematic Review and Meta-Analysis

**DOI:** 10.3390/ijerph18062912

**Published:** 2021-03-12

**Authors:** Hyun Suk Lee, Junga Lee

**Affiliations:** 1 Graduate School of Education, Chung-Ang University, Seoul 06974, Korea; hslee@cau.ac.kr; 2 Sports Medicine and Science, Kyung Hee University, Gyeonggi-do 17104, Korea

**Keywords:** athletes, ketogenic diet, meta-analysis

## Abstract

(1) Background: The purpose of the current meta-analysis was to investigate any positive or negative effects of ketogenic diets in athletes and provide an assessment of the size of these effects. (2) Methods: Databases were used to select relevant studies up to January 2021 regarding the effects of ketogenic diets in athletes. Inclusion criteria were as follows: data before and after ketogenic diet use, being randomized controlled trials and presenting ketogenic diets and assessments of ketone status. Study subjects were required to be professional athletes. Review studies, pilot studies, and studies in which non-athletes were included were excluded from this meta-analysis. The outcome effect sizes in these selected studies were calculated by using the standardized mean difference statistic. (3) Results: Eight studies were selected for this meta-analysis. Athletes who consumed the ketogenic diet had reduced body fat percentages, respiratory exchange rates, and increased total cholesterol compared to athletes who did not consume this diet. However, body mass index, cardiorespiratory fitness, heart rate, HDL cholesterol, glucose level, and insulin level were unaffected by the diet. (4) Conclusions: Ketogenic diets had a beneficial effect by decreasing body fat percentage, but athletes with high total cholesterol level need to be monitored when instituting a ketogenic diet. Our study sample size was limited; therefore, additional studies may be needed to confirm the current findings. Further studies need to be conducted on changes in LDL cholesterol, HDL cholesterol, total cholesterol and ratio of LDL to HDL cholesterol.

## 1. Introduction

Athletes who require weight control for participation in weight category sports or athletes attempting to improve performance are interested in ketogenic diets. A ketogenic diet consists of a low level of carbohydrates (less than 50 g/day) relative to intakes of calories and fat [1]. Several ketogenic studies suggested that the beneficial effects of ketogenic diets included reduced weight; reduced fasting glucose and insulin levels; less exercise-induced oxidative stress and inflammation; improved cognition and mood; decreased fat oxidation, and sparing of glycogen stores [2,3]. The reductions in total carbohydrate intake with low glycemic index diets were also suggested to decrease cardiometabolic risk [4].

Several ketogenic diet studies have been reported in athletes to assess improvement in submaximal intensity exercise [1,5]. Increased fat oxidation after a ketogenic diet affected athletic performance [3,5,6]. However, there were several questions on whether or not the ketogenic diet had any positive or negative influence on athletic body composition, exercise capacity, and physiological factors. These factors included heart rate, insulin level, and levels of cholesterols; the effects of ketogenic diets on these factors in athletes were inconsistent. A previous study reported a negative effect of ketogenic diet on athletic endurance performance [7]. The reasoning was that alterations in the main energy sources from carbohydrate to fat oxidation limited glycogen stores, leading to restrictive muscle protein accumulations. A previous review study reported no clear benefits of ketogenic diets on athletic performance. However, some short duration benefits on vigorous intensity test outcomes were reported [5]. This review study did not calculate the effect sizes of ketogenic diets on athletic performance.

One main consideration was the effect of ketogenic diets on lipid profiles. Some previous studies reported decreased total cholesterol, LDL cholesterol, and triglycerides; other studies did not find changes in those variables after completion of ketogenic diets [2,6,7]. Lipid profile assessments were crucial because increased lipid profiles are associated with increased risk of cardiometabolic diseases that include heart disease [8], and the link between lipid markers and heart disease is complex [9]. Additionally, ketogenic diet influences on athletes’ weight, fat level, heart rate, glucose level, and insulin level needed to be identified. Understanding the effects of ketogenic diet on those outcomes in an athletic group can help not only to expand aerobic and resistance training in athletes, but also to address controversial findings including cardiometabolic and physiological factors and lipid profiles. While levels of physical fitness and exercise need to be controlled in ketogenic diet studies, limited studies have restricted physical fitness and exercise. A more heterogeneous pool of subjects including an athletic group will increase understanding on the effects of a ketogenic diet. Therefore, the purpose of this meta-analysis was to investigate the positive and negative effects of ketogenic diets on body composition; cardiometabolic factors including respiratory exchange rate; cardiorespiratory fitness and heart rate; lipid profiles including total cholesterol, LDL cholesterol, and HDL cholesterol; and physiological factors including glucose and insulin levels by reviewing all relevant studies. We calculated the size of these effects on athletic performance.

## 2. Materials and Methods

### 2.1. Searching Processes to Identify Eligible Studies

EMBASE and MEDLINE databases were used to find relevant studies. All studies were published in the English language up to October, 2020. Search terms for this meta-analysis were “athletes” (athletic, sports, players) AND “ketogenic diet” (low carbohydrate and high fat). All possible combinations of the search terms were used to select relevant studies. One researcher (J. L.) and one reviewer (H. L.) independently selected relevant studies based on inclusion and exclusion criteria. Further discussions resolved any disagreements in the selection processes. The citation lists of review studies were screened to find any relevant studies missed. Inclusion criteria for this meta-analysis were randomized control trials that reported outcomes of ketogenic diet use in athletes. Athletes must have comprised both experimental and control groups. Descriptions of the ketogenic diet and assessments of ketone status were required. Pilot and review studies and studies of non-athlete populations were excluded. The Cochrane Collaboration’s Risk of Bias Tool was used to assess quality and risk of bias [10]. This tool is a seven-domain bias assessment: random sequence generation, allocation concealment, blinding of participants and personnel, blinding of outcomes, incomplete outcome data, selective reporting, and other biases. These domains provided an assessment of bias and overall quality of the reports. Cut-off points or scores were not indicated in the assessment. Two researchers (J. L and H. L.) also evaluated the quality and bias independently. Additional discussions resolved any disagreements.

### 2.2. Statistical Analysis

Effect sizes of ketogenic diet interventions for athletes were calculated by using the standardized mean difference statistic, the difference in both treatment and control groups’ means divided by the pooled standard deviation. If there were more than two outcomes, the effect size was computed. Effect sizes between 0.2 and 0.5 were considered to be small. A medium effect size was 0.5–0.8 and a large effect size was >0.8. The Q statistic was used to determined heterogeneity (*p* = 0.1). If I^2^ was ≤50%, heterogeneity was absent and a fixed-effect model was used. If I^2^ was >50%, heterogeneity existed and a random-effects model was used. Statistical significance was set at *p* < 0.05. All analyses were conducted using Comprehensive Meta-Analysis Version 1.25 software (Biostatic Inc., Englewood, NJ, USA).

## 3. Results

**Figure 1:**
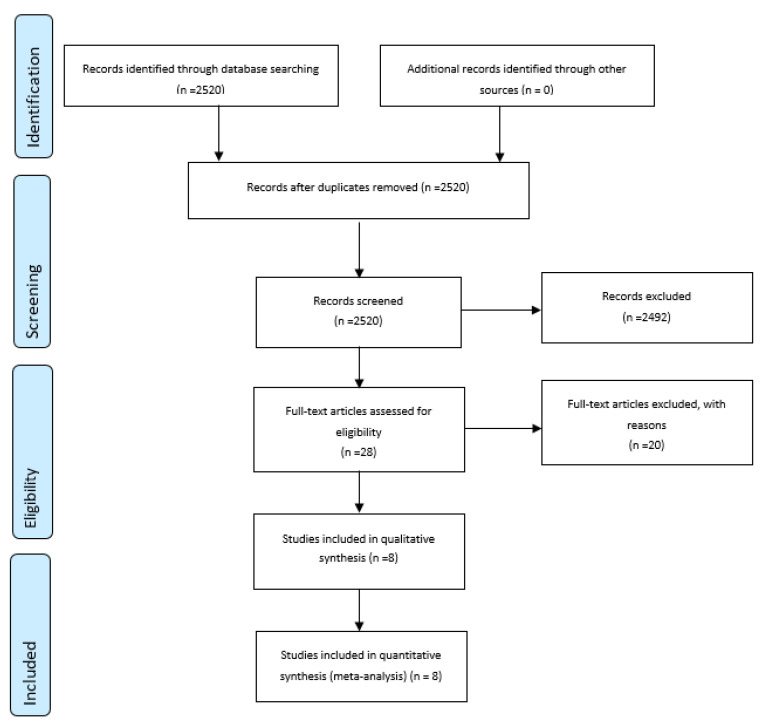
Selection process for the systematic review and meta-analysis.

The selection process for the systematic review and meta-analysis is presented in Figure 1. Those search terms were used for initial screening, and 540 studies were retrieved in the initial screening. All titles and abstracts were used to determine the eligibility for inclusion in the meta-analysis; and 453 studies that were review studies, pilot studies, protocol studies, or recreationally trained individual studies, and studies that were not related to ketogenic diets and exercise were excluded. The remaining 87 studies’ full texts were reviewed to determine eligibility. An additional 80 studies were excluded. These did not include pre- or post-ketogenic diet outcome data, were not restricted to professional athlete and control groups, or did not use a standard diet. Therefore, eight studies [11,12,13,14,15,16,17,18] were selected for the current meta-analysis. The average ketogenic diet was 3 weeks and ranged from 1 week to 24 weeks. During ketogenic diet use, the athletes participated in their training. Ketone status was monitored. Aerobic athletes were included in four studies, and resistance-trained athletes were included in four studies. 

Basic characteristics of the selected studies, including name of the first author, year of publication, country in which the study was conducted, design of the study, sample size, participants’ ages and sports involved, ketogenic diet processes and ketone assessments, and main outcomes, are presented in Table 1. The quality and bias of risk measured by the Cochrane Collaboration’s Risk of Bias Tool is presented in Supplementary Table S1. A high degree of bias was not detected in any of the selected studies. Each common outcome variable was used to calculate the effect size when the outcome variable was reported in at least two studies. All outcomes of the selected studies were included to calculate effect size.

### 3.1. Influences of Ketogenic Diet on Body Composition

**Figure 2:**
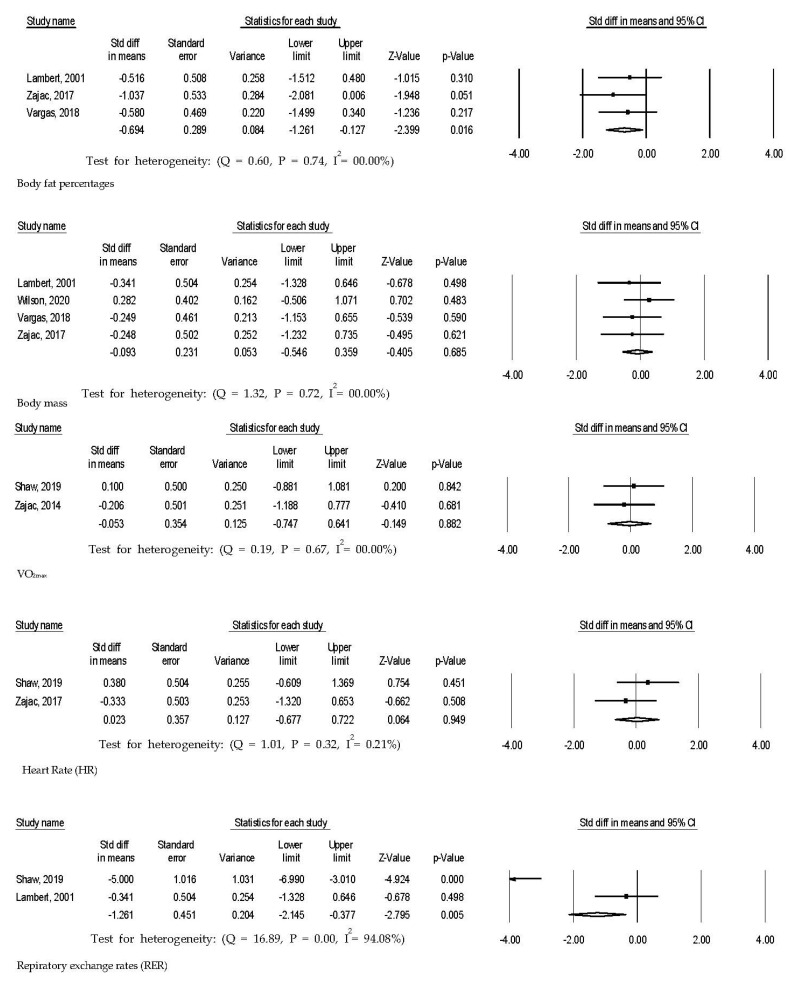

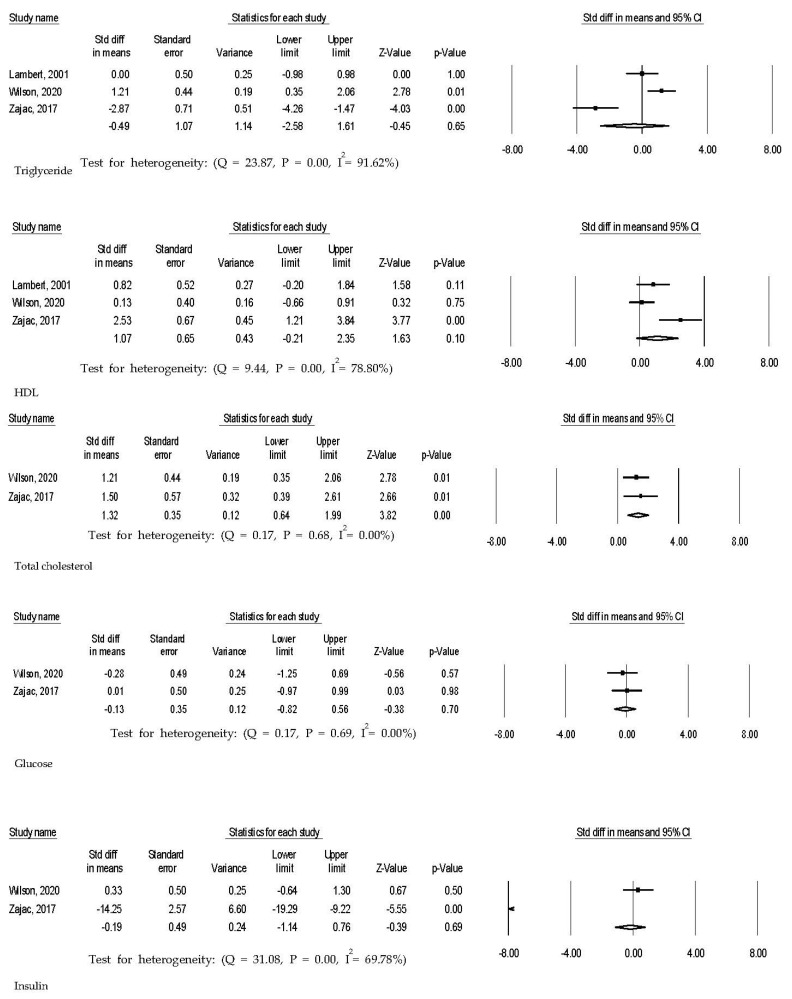
Effect sizes of ketogenic diet interventions for athletes.

Four trials that tested % body fat were included to calculate effect size (Figure 2). Athletes who participated in the ketogenic diet had statistically significant reduced body fat percentages (d = −0.48 [95% confidence interval, −0.96–0.00; *p* = 0.05; k = 4]) compared to control athletes who consumed non-ketogenic diets. However, those who consumed ketogenic diets did not have statistically significant changes in body mass (d = 0.05 [95% confidence interval, −0.32–0.42; *p* = 0.77; k = 4]) compared to those of non-ketogenic diet athletes.

### 3.2. Influences of Ketogenic Diet on Respiratory Exchange Rate at VO_2max_, Cardiorespiratory Fitness, and Heart Rate

Those on ketogenic diets in three trials demonstrated statistically significantly reduced respiratory exchange rates (d = −1.92 [95% confidence interval, −2.70–−1.14; *p* = 0.01; k = 3]) compared to control groups (Figure 2). However, there were no statistical differences in VO_2max_ (d = 0.12 [95% confidence interval, −0.35–0.59; *p* = 0.62; k = 4]) in four trials and heart rate (d = −0.48 [95% confidence interval, −0.96–0.00; *p* = 0.44; k = 3]) in three trials between experimental and control groups.

### 3.3. Influences of Ketogenic Diet on Total Cholesterol, HDL Cholesterol, Triglyceride, Glucose Level, and Insulin Level

Total cholesterol in athletes consuming ketogenic diets was statistically significantly reduced (d = 1.32 [95% confidence interval, 0.64–1.99; *p* = 0.05; k = 2]) in two trials (Figure 2). However, HDL cholesterol (d = 1.07 [95% confidence interval, −0.21–2.35; *p* = 0.10; k = 3]) in three trials, triglyceride (d = −0.49 [95% confidence interval, −2.58–1.61; *p* = 0.65; k = 3]) in three trials, glucose level (d = −0.13 [95% confidence interval, −0.82–0.56; *p* = 0.70; k = 2]) in two trials and insulin level (d = −0.19 [95% confidence interval, −0.14–0.76; *p* = 0.69; k = 2]) in two trials did not statistically significantly change compared to control athletes.

## 4. Discussion

Professional athletes who consumed ketogenic diets had reduced body fat percentages, increased respiratory exchange rates, and increased total cholesterol levels. All participants exercised, but diet was the only difference between the experimental groups and the control groups. Ketogenic diets were consumed for an average of 10 weeks, and average exercise interventions were moderate to vigorous exercise interventions five times per week. The ketogenic diets may help to reduce body fat percentages when professional athletes need to decrease body mass; however, before starting the ketogenic diet, total cholesterol level should be monitored in those with high cholesterol levels at baseline.

The ketogenic diets were effective in reducing body fat percentages of professional athletes. Additionally, the ketogenic diet groups had increased VO_2max_ that indicated levels of cardiorespiratory fitness, but these measurements did not reach statistical significance. There were no meta-analysis studies that compared those results. A previous pilot study demonstrated reduced body weight, body fat, and well-being, but not exercise performance, in endurance athletes after completing ketogenic diets [19]. Another previous study demonstrated that the VO_2max_ had a negative correlation with body fat percentage in female athletes but it was not statistically significant [20]. While additional studies are needed to support the effects of ketogenic diets, athletes who consumed ketogenic diets had reduced body fat percentages, which may be associated with increased aerobic capacity.

Athletes who consumed ketogenic diets had a statistically significantly decreased respiratory exchange rate on the VO_2max_ test, but cardiorespiratory fitness and heart rate did not change significantly. The reduced respiratory exchange rate after consuming ketogenic diets was consistent with the findings of a previous study [21]. Increased high fat intakes were associated with reduced respiratory exchange rate [22]. Changes in cardiorespiratory fitness findings are controversial, but we did not find statistically significant changes in our meta-analysis. Previous studies demonstrated impairments in high intensity, endurance performance after ketogenic diet adaption [3,10,16] due to reduced glycogenolysis and prorate dehydrogenase activity [23] and increased heart rate [11] that was associated with increased sympathetic nervous system activity [24]. Other studies did not demonstrate statistically significant changes in cardiorespiratory fitness and heart rate [15]. Respiratory exchange rate, cardiorespiratory fitness and heart rate were influenced by exercise type in both aerobic athletes and resistance athletes when the athletes consumed the ketogenic diet. The findings on exercise type may need to be confirmed in further studies.

When athletes consumed ketogenic diets during exercise training, these athletes had increased total cholesterol compared to control groups. Although total cholesterol has become an irrelevant isolated marker for heart disease risk for any population [25,26], athletes with pre-existing high level of total cholesterol should be monitored while on a ketogenic diet. Dietary macronutrient composition was not statistically significantly different between the two groups, but athletes had significantly higher energy intake and energy expenditure compared to non-athletes [27]. The lipid profiles may be not influenced by training or non-training, but the nutrient composition effects on lipid profile levels included total cholesterol, HDL cholesterol, and LDL cholesterol. Several studies demonstrated that participating in exercise decreased lipid profiles including total cholesterol, LDL cholesterol, triglycerides, and increased HDL cholesterol [28,29], but a study of athletes professionally or recreationally trained had conflicting results after participants completed ketogenic diet use. A previous study reported increased LDL cholesterol, but decreased body fat, in recreationally trained CrossFit trainees after participating in ketogenic diet use [30]. Another previous study found no statistically significant changes in athlete lipid profiles [17]. Although additional studies are needed to confirm lipid profile results, the lipid profiles were associated with diverse factors including family history and nutritional habits. Additionally, the effect size of lipid profile as measured by LDL cholesterol and ratio of HDL cholesterol to LDL cholesterol was not investigated due to limited numbers of studies or missing data. Insulin and glucose levels were decreased in athletes using ketogenic diets, but the results in the current studies were not statistically significant. The restricted source of glucose removed the trigger of insulin release that led to decreased insulin levels [31]. The effects of ketogenic diets in athletes on insulin and glucose levels need further investigation.

There were several limitations in the current meta-analysis. First, while effect size was calculated for two trials, sample sizes were small. Caution needs to be exercised in generalizing these results. Second, professional athletes who performed both endurance and resistance exercise were included. Depending on the exercise type, the results of this meta-analysis will need recalculation when additional studies regarding the effects of ketogenic diets on athletes are added. Third, the periods of ketogenic diets and exercise interventions were diverse; this may have had an influence on the outcomes. Both short-term and long-term use of ketogenic diet and exercise interventions need to be conducted. Fourth, variables of total cholesterol, HDL cholesterol, triglycerides, and glucose were one-off measures that have the possibility of over- or underestimation of results. The LDL data that are an important assessment of cardiometabolic risk were not reported in studies that limited current findings to the effects of ketogenic diet on lipid profiles. More studies about the ketogenic diet and lipid profiles are needed. Last, all selected studies assessed ketone status, but the levels of ketones and assessment tools for the ketone status were not consistent across all selected studies.

There are potential mechanisms of the ketogenic diet on reducing body fat percentage among athletes. Increased intake of fat in daily meals elevated total fat oxidation during exercise [1,32]. Additionally, a higher level of fat oxidation shifted utilization from carbohydrates and led to concomitant muscle glycogen sparing [33,34]. Increased fat oxidation might be associated with the decreased body fat percentage observed in athletes on a ketogenic diet compared to those on a standard diet.

## 5. Conclusions

Ketogenic diets reduced body fat percentages and increased respiratory exchange rates and total cholesterol. Additional studies may be needed to confirm these results. The athletes in this current meta-analysis study participated in an average of five weeks of ketogenic diet consumption. Additional studies on the shorter- and longer-term effects of ketogenic diets on athlete body composition, cardiometabolic factors including respiratory exchange, cardiorespiratory fitness and heart rate, and lipid profiles may be required. To further understand the effects of ketogenic diets on athletes, exercise type may also need to be considered in future studies.

## Figures and Tables

**Table 1:** Basic Characteristics of selected studies.

First Author (Year), Country	Design, Number of Participants per Group, Age (Years Old)	Participants/Control	Exercise Intervention	Ketogenic Diet including Length, Energy, and Compositions/Contorl Group/Assessments of Ketosis	Outcomes
Aerobic exercise					
Goedecke, (1999), South Africa [13]	Randomized controlled trails: high fat diet (HFD, *n* = 8, 24 ± 3 years old) vs. control (CON, *n* = 8, 30 ± 9 years old)	Endurance-trained male cyclists	4 weeks, high intensity interval training 10 × 60 s cycling intervals & 60 s of recovery, 3 days/week	15 days, no restricted calories, HFD: total energy (4334 ± 3382 kJ), fat (32 ± 8%), carbohydrate (48 ± 10%), protein (14 ± 2%), control: 13,210 ± 3382 kJ, fat (30 ± 8%), carbohydrate (53 ± 10%), protein (13 ± 3%), blood samples	Glucose, lactate, insulin, free fat oxidation, β-hydroxybutyrate, plasma glucose oxidation, ingested glucose oxidation, muscle glycogen oxidation, carnitine acyltransferase (CAD), 3-hydrozyacyl-coenzyme a dehydrogenase (3-HAD), citrate synthase (CS), glucose transport-4 (GLUT-4)
Lambert (2001), United Kingdom [15]	Randomized cross over trails: high fat (HF, *n* = 8) vs. control (*n* = 8), average 22.4 ± 1.5 year old	Endurance-trained male cyclists	4 weeks, high intensity interval training 10 × 60 s cycling intervals & 60 s of recovery, 3 days/week	14 days, high fat (energy 14.00 MJ, carbohydrate: <15%, protein: 20%, fat: >65%), habitual diet (energy 14.3 ± 0.7 MJ, carbohydrate: 52.6 ± 5.8, protein: 12.8 ± 0.9, fat: 29.9 ± 4.5), ß-hydroxybutyrate ketone level	Weight, body fat, serum cholesterol, serum high density lipoprotein (HDL) cholesterol, serum triglyceride
Zajac, (2014), Australia [18]	Randomized cross over trails: exercise + low carbohydrate diet (*n* = 8, 25.4 ± 4.0 years old) vs. control (*n* = 8, 25 ± 7 years old)	Cyclists, body fat	6 months, ≥30 min/day, ≥5 days/week	6 months, 2200 kcal/day, isocaloric diets in keteogenic diet and standard Western diet, ketogeic diet (70% fat, 15% protein, 15% carbohydrates), mixed or standard Western diet (50% carbohydrates, 30% fat, 20% protein), 30 ß-hydroxybutyrate ketone level	Carbohydrates, fat, protein, calories, weight, Body mass index, HOMA, glucose, insulin, HbA1c, cholesterol, low density lipoprotein (LDL) cholesterol, HDL cholesterol, triglycerides, prostate-specific antigen, HsCRP, heart rate, VO_2max_, respiratory exchange rate (RER)
Burke (2017), Australia [12]	Randomized controlled trails: high carbohydrate diet (HCD, *n* = 9, 25.4 ± 4.0 years old), periodised carbohydrate (PCHOD, *n* = 10, 27.4 ± 4.6 years old) low carbohydrate high fat diet (LCHFD, *n* = 10, 28.3 ± 3.5 year old)	Elite race walkers	4 days, ~30 min walking after breakfast, lunch, and dinner, ~60% of maximal heart rate, accelerometer	3 weeks, HC: daliy energy intake: 14.73 MJ 231 kJ/kg (carbohydrate: 549 g, 8.6 g/kg, 60% energy, protein:138 g, 2.1 g/kg, 16% energy, fat: 77 g, 1.2 g/kg, 20% energy), PCHO: daliy energy intake: 14.89 MJ 226 kJ/kg (fat: 79 g, 1.2 g/kg, 20%, protein: 144 g, 2.2 g/kg, 17% energy, carbohydrate:547g, 8.3 g/kg, 60% enegry), LCHF: daliy energy intake: 14.90 MJ 223 kJ/kg (fat: 312 g, 4.7 g/kg, 78% energy, protein: 144 g, 2.2 g/kg, 17% energy, carbohydrate:33g, 0.5g/kg, 3.5% enegry), ß-hydroxybutyrate ketone level	Body mass, respiratory exchange ratio, VO_2max_, heart rate, perceived exertion (RPE)
Resistance exercise					
Wilson (2017), U.S.A. [17]	Randomized controlled trails: exercise + ketogenic diet (*n* = 13, 23.5 ± 4.5 years old), vs. exercise + regular diet (*n* = 12, 21.3 ± 3.7 years old)	Resistance training men	11 weeks, resistance exercise (week 1: 65% and 15 repetitions, 85% and 5 repetitions, week 2: 70% and 12 repetitions, 90% and 4 repetitions, week 3: 75% and 10 repetitions, 90% and 3 repetitions week 4: 77.5% and 8 repetitions, 90–100% and 1–2 repetitions, week 3–6 hypertrophy, week 7–10: strength, squat, lunges, leg extension, leg curl, bench, stiff leg deadlift, hyperextension, leg press, calf press)	10~11 weeks, ketogenic diet: 2652.9 ± 205.6 kcal/day, 75% fat, 20% protein, 5% carbohydrate, until blood ketone bodies detected, regular diet: 2528.1 ± 200.4 kcal/day, 20% protein, 5% carbohydrate, 75% fat	Total mass, muscle thickness, bench press, squat, Wingate power, testosterone total, testosterone free, inclusion blood glucose, triglycerides, total cholesterol, high density lipoprotein
Greene (2018), Australia [14]	Randomized cross over trails: low carbohydrate ketogenic diet (*n* = 12), vs. usual diet (*n* = 12), average 35 ± 5 years old	Elite powerlifters and Olympic weight lifters	4 weeks, high intensity interval training (10 × 6 s cycling sprints & 9 s recovery, total 2.5 min/session), moderate intensity continuous training (30 min cycling, 5 min for warm-up, 50% of VO_2peak_ for 10 min, 60% of VO_2peask_ for 10 min, 5 min for recovery), 5 days/week, pedometers, logbook	12 weeks, low carbohydrate ketogenic diet: usal diet diet: [male (energy intake: 9868.1 ± 1322.9 kJ/day, carbohydrate: 7.5 ± 2.1%, fat: 70.1 ± 6.6%, protein: 22.4 ± 5.7%, female (energy intake: 6995.5 ± 1554.5 kJ/day, carbohydrate: 8.9 ± 1.7%, fat: 67.6 ± 4.1%, protein: 23.5 ± 2.9%, usal diet diet: [male (energy intake: 9653.2 ± 1885.4 kJ/day, carbohydrate: 44.7 ± 2.8%, fat: 33.7 ± 6.2%, protein: 21.7 ± 6.5%, female (energy intake: 7147.5 ± 1500 kJ/day, carbohydrate: 45.1 ± 7.1%, fat: 32.4 ± 6.3%, protein: 22.5 ± 5.8%, ß-hydroxybutyrate ketone level	Body mass, fat mass, lean mass, 1 repetition maximum (1RM), resting metabolic rate (RMR), respiratory quotient, glucose, potassium, sodium
Vargas (2018), Spain [16]	Randomized controlled trails: exercise + ketogenic diet (*n* = 9), non-ketogenic diet (*n* = 10) vs. control (*n* = 5), average 30 ± 4.7 years old	Trained men	8 weeks, hypertrophy training (upper limb and lower limb 2 times per week)	8 weeks, a daily energy intake ≥39 kcal/kg/day, ketogenic diet: 70% fat, 20% protein, <10% carbohydrate, and until blood ketone were monitored weekly, non-ketogenic diet: 55% carbohydrat, 20% fat, 20% protein	Body weight, fat mass, visceral adipose tissue, lean body mass
Shaw (2019), New Zealand, [11]	Randomized repeated-measure crossover study, CT: exercise + ketogenic diet (*n* = 8), average 29.6 ± 5.1 uears old	Trained male endurance athletes	28 days, running and cycling training	28 days, ketogenic diet: [male (energy intake: 13.73 ± 2.43 MJ/day, carbohydrate: 4.1 ± 0.8%, fat: 77.7 ± 3.9%, protein: 18.2 ± 3.5%, usal diet diet: [male (energy intake: 13.07 ± 1.67 MJ/day, carbohydrate: 42.9 ± 7.8%, fat: 38.5 ± 7.1%, protein: 18.6 ± 1.45%, ß-hydroxybutyrate ketone level	Body mass, VO_2_max (L∙min^−1^), respiratory exchange ratio, energy expenditure, heart rate, rate of perceived exertion

HOMA = homeostatic model assessment; VO_2max_ = maximal oxygen uptake.

## Supplementary Materials

The following are available online at https://www.mdpi.com/1660-4601/18/6/2912/s1, Table S1: Assessments of the quality and risk of bias title.

## References

[1] Phinney S.D., Bistrian B.R., Evans W.J., Gervino E., Blackburn G.L. (1983). The human metabolic response to chronic ketosis without caloric restriction: Preservation of submaximal exercise capability with reduced carbohydrate oxidation. Metabolism.

[2] Noakes T., Volek J.S., Phinney S.D. (2014). Low-carbohydrate diets for athletes: What evidence?. Br. J. Sports Med..

[3] Rhyu H.S., Cho S.Y. (2014). The effect of weight loss by ketogenic diet on the body composition, performance-related physical fitness factors and cytokines of Taekwondo athletes. J. Exerc. Rehabil..

[4] Paoli A., Bianco A., Grimaldi K.A. (2015). The Ketogenic Diet and Sport: A Possible Marriage?. Exerc. Sport Sci. Rev..

[5] McSwiney F.T., Doyle L., Plews D.J., Zinn C. (2019). Impact Of Ketogenic Diet On Athletes: Current Insights. Open Access J. Sports Med..

[6] Durkalec-Michalski K., Nowaczyk P.M., Siedzik K. (2019). Effect of a four-week ketogenic diet on exercise metabolism in CrossFit-trained athletes. J. Int. Soc. Sports Nutr..

[7] Kiens B., Helge J.W. (1998). Effect of high-fat diets on exercise performance. Proc. Nutr. Soc..

[8] Micha R., Mozaffarian D. (2010). Saturated fat and cardiometabolic risk factors, coronary heart disease, stroke, and diabetes: A fresh look at the evidence. Lipids.

[9] Volek J.S., Freidenreich D.J., Saenz C., Kunces L.J., Creighton B.C., Bartley J.M., Davitt P.M., Munoz C.X., Anderson J.M., Maresh C.M. (2016). Metabolic characteristics of keto-adapted ultra-endurance runners. Metabolism.

[10] Higgins J.P., Altman D.G., Gotzsche P.C., Juni P., Moher D., Oxman A.D., Savovic J., Schulz K.F., Weeks L., Sterne J.A. (2011). The Cochrane Collaboration’s tool for assessing risk of bias in randomised trials. BMJ.

[11] Shaw D.M., Merien F., Braakhuis A., Maunder E.D., Dulson D.K. (2019). Effect of a Ketogenic Diet on Submaximal Exercise Capacity and Efficiency in Runners. Med. Sci. Sports Exerc..

[12] Burke L.M., Ross M.L., Garvican-Lewis L.A., Welvaert M., Heikura I.A., Forbes S.G., Mirtschin J.G., Cato L.E., Strobel N., Sharma A.P. (2017). Low carbohydrate, high fat diet impairs exercise economy and negates the performance benefit from intensified training in elite race walkers. J. Physiol..

[13] Goedecke J.H., Elmer-English R., Dennis S.C., Schloss I., Noakes T.D., Lambert E.V. (1999). Effects of medium-chain triaclyglycerol ingested with carbohydrate on metabolism and exercise performance. Int. J. Sport Nutr..

[14] Greene D.A., Varley B.J., Hartwig T.B., Chapman P., Rigney M. (2018). A Low-Carbohydrate Ketogenic Diet Reduces Body Mass Without Compromising Performance in Powerlifting and Olympic Weightlifting Athletes. J. Strength Cond. Res..

[15] Lambert E.V., Goedecke J.H., Zyle C., Murphy K., Hawley J.A., Dennis S.C., Noakes T.D. (2001). High-fat diet versus habitual diet prior to carbohydrate loading: Effects of exercise metabolism and cycling performance. Int. J. Sport Nutr. Exerc. Metab..

[16] Vargas S., Romance R., Petro J.L., Bonilla D.A., Galancho I., Espinar S., Kreider R.B., Benitez-Porres J. (2018). Efficacy of ketogenic diet on body composition during resistance training in trained men: A randomized controlled trial. J. Int. Soc. Sports Nutr..

[17] Wilson J.M., Lowery R.P., Roberts M.D., Sharp M.H., Joy J.M., Shields K.A., Partl J.M., Volek J.S., D’Agostino D.P. (2020). Effects of Ketogenic Dieting on Body Composition, Strength, Power, and Hormonal Profiles in Resistance Training Men. J. Strength Cond Res..

[18] Zajac A., Poprzecki S., Maszczyk A., Czuba M., Michalczyk M., Zydek G. (2014). The effects of a ketogenic diet on exercise metabolism and physical performance in off-road cyclists. Nutrients.

[19] Zinn C., Wood M., Williden M., Chatterton S., Maunder E. (2017). Ketogenic diet benefits body composition and well-being but not performance in a pilot case study of New Zealand endurance athletes. J. Int. Soc. Sports Nutr..

[20] Shete A.N., Bute S.S., Deshmukh P.R. (2014). A Study of VO2 Max and Body Fat Percentage in Female Athletes. J. Clin. Diagn. Res..

[21] McSwiney F.T., Fusco B., McCabe L., Lombard A., Crowley P., Walsh J., Hone M., Egan B. (2021). Changes in body composition and substrate utilization after a short-term ketogenic diet in eundurance-trained males. Biol. Sport.

[22] Lambert E.V., Wooding G., Lambert M.I., Koeslag J.H., Noakes T.D. (1994). Enhanced adipose tissue lipoprotein lipase activity in detrained rats: Independent of changes in food intake. J. Appl. Physiol..

[23] Stellingwerff T., Spriet L.L., Watt M.J., Kimber N.E., Hargreaves M., Hawley J.A., Burke L.M. (2006). Decreased PDH activation and glycogenolysis during exercise following fat adaptation with carbohydrate restoration. Am. J. Physiol. Endocrinol. Metab..

[24] Havemann L., West S.J., Goedecke J.H., Macdonald I.A., St Clair Gibson A., Noakes T.D., Lambert E.V. (2006). Fat adaptation followed by carbohydrate loading compromises high-intensity sprint performance. J. Appl. Physiol..

[25] Halldin A.K., Lissner L., Lernfelt B., Bjorkelund C. (2020). Cholesterol and triglyceride levels in midlife and risk of heart failure in women, a longitudinal study: The prospective population study of women in Gothenburg. BMJ Open.

[26] Canoui-Poitrine F., Luc G., Bard J.M., Ferrieres J., Yarnell J., Arveiler D., Morange P., Kee F., Evans A., Amouyel P. (2010). Relative contribution of lipids and apolipoproteins to incident coronary heart disease and ischemic stroke: The PRIME Study. Cerebrovasc. Dis..

[27] Petridou A., Lazaridou D., Mougios V. (2005). Lipidemic profile of athletes and non-athletes with similar body fat. Int. J. Sport Nutr. Exerc. Metab..

[28] Marques E., Carvalho J., Soares J.M., Marques F., Mota J. (2009). Effects of resistance and multicomponent exercise on lipid profiles of older women. Maturitas.

[29] Gordon L.A., Morrison E.Y., McGrowder D.A., Young R., Fraser Y.T., Zamora E.M., Alexander-Lindo R.L., Irving R.R. (2008). Effect of exercise therapy on lipid profile and oxidative stress indicators in patients with type 2 diabetes. BMC Complement Altern Med..

[30] Kephart W.C., Pledge C.D., Roberson P.A., Mumford P.W., Romero M.A., Mobley C.B., Martin J.S., Young K.C., Lowery R.P., Wilson J.M. (2018). The Three-Month Effects of a Ketogenic Diet on Body Composition, Blood Parameters, and Performance Metrics in CrossFit Trainees: A Pilot Study. Sports.

[31] Kinzig K.P., Honors M.A., Hargrave S.L. (2010). Insulin sensitivity and glucose tolerance are altered by maintenance on a ketogenic diet. Endocrinology.

[32] Helge J.W., Richter E.A., Kiens B. (1996). Interaction of training and diet on metabolism and endurance during exercise in man. J. Physiol..

[33] Bergstrom J., Hermansen L., Hultman E., Saltin B. (1967). Diet, muscle glycogen and physical performance. Acta Physiol. Scand..

[34] Jansson E., Kaijser L. (1982). Effect of diet on muscle glycogen and blood glucose utilization during a short-term exercise in man. Acta Physiol. Scand..

