# Supplementary material for: Influences of Ketogenic Diet on Body Fat Percentage, Respiratory Exchange Rate, and Total Cholesterol in Athletes: A Systematic Review and Meta-Analysis
Source: Int J Environ Res Public Health. 2021 Mar 12;18(6):2912. doi: 10.3390/ijerph18062912 (PMC7999937; doi:10.3390/ijerph18062912)
Supplement: Supplementary file 1 [file ijerph-18-02912-s001.pdf]

Supplementary Table S1. Assessments of the quality and risk of bias							
First Author (year)	Random sequence generation	Allocation concealment	Blinding of participants and personnel	Blinding of outcome assessment	Incomplete outcome data	Selective reporting	Other bias
Goedecke, (1999), South Africa	+	+	?	?	+	?	+
Lambert (2001), United Kingdom	+	-	-	-	+	?	+
Zajac, (2014), Australia	+	-	-	-	+	?	+
Burke (2017), Australia	+	+	?	?	+	?	+
Wilson (2017), U.S.A.	+	+	?	?	+	?	+
Greene (2018), Australia	+	-	-	-	+	?	+
Vargas (2018), Spain	+	+	?	?	+	?	+
Shaw (2019), New Zealand	+	-	-	?	+	?	+

+ = Low risk of bias, - = High risk of bias, ? = Unclear risk of bias
